# Supplementary material for: The complexity of the stream of consciousness
Source: Commun Biol. 2022 Nov 3;5:1173. doi: 10.1038/s42003-022-04109-x (PMC9633704; doi:10.1038/s42003-022-04109-x)
Supplement: Supplementary file 2 — Supplementary Information-New [file 42003_2022_4109_MOESM2_ESM.pdf]

Supplementary Notes for

The complexity of the stream of consciousness

Peter Coppola, Judith Allanson, Lorina Naci, Ram Adapa, Paola Finoia, Guy B. Williams, John D. Pickard, Adrian M. Owen, David K. Menon & Emmanuel A. Stamatakis*.

Supplementary Note 1

Parcellation Description

Timeseries were extracted using several atlases to confirm results were not dependent on specific brain region definitions. To attain whole brain coverage, the Schaefer cortical parcellations (Schaefer et al., 2018) were united with the Melbourne subcortical atlas (Tian, Margulies, Breakspear, & Zalesky, 2020) and a cerebellar atlas (Ren, Guo, & Guo, 2019). These parcellations are characterised by the availability of different granularities, thus enabling a whole brain atlas that has similar ROI sizes across subsystems. These parcellations were all derived using functional connectivity in a data-driven manner (Ren et al., 2019; Schaefer et al., 2018; Tian et al., 2020).

We merged these Cortical, Subcortical and Cerebellar atlases to create whole brain parcellations with 126 (WB126) and 553 regions (WB553).

Table S1. Composition of Whole Brain Parcellations (WB126 & WB553)

Parcellations	Cortical (Schaefer et al., 2018)	Subcortical (Tian et al., 2020)	Cerebellar (Ren et al., 2019)
WB126	100	16	10
WB553	400	54	99

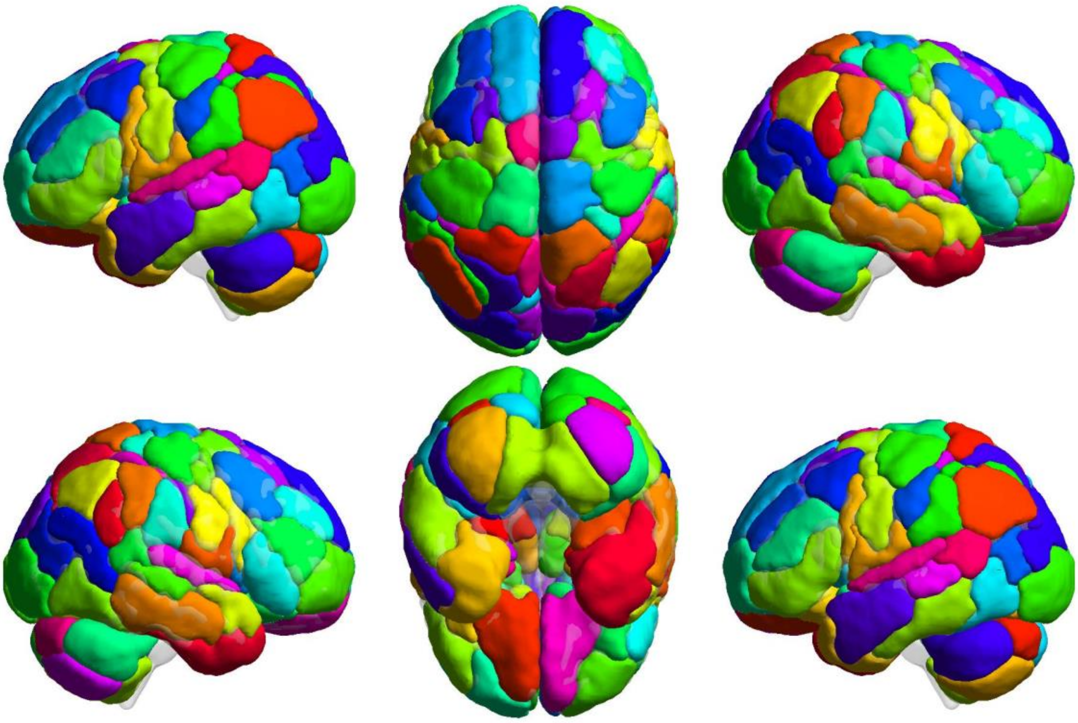

Figure S1. Whole Brain Parcellation -126 parcels

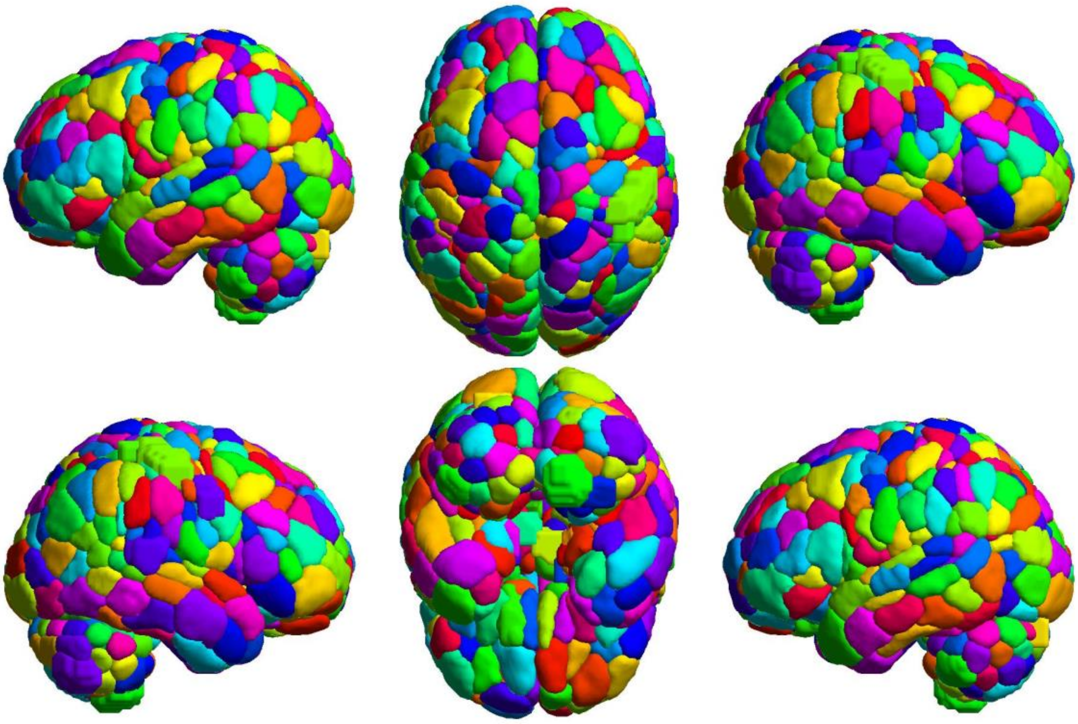

Figure S2. Whole Brain Parcellation – 553 parcels

Links for download

Schaefer Cortical Atlas : https://github.com/ThomasYeoLab/CBIG/tree/master/stable_projects/brain_parcellation/Schaefer2018_LocalGlobal

Melbourne Subcortical Atlas : https://github.com/yetianmed/subcortex/blob/master/Group-Parcellation/3T/Subcortex-Only/Tian_Subcortex_S4_3T_label.txt

Cerebellar Atlas : http://neuroguo.com/resources/

The Cerebellar and cortical parcellations had some overlapping voxels (154 voxels for both WB126 and WB553). We removed these voxels from the cerebellar atlas to ensure no repeated voxels between different regions of interest (ROIs) in the whole brain atlas'.

Supplementary Note 2

Display and discussion of different methods to obtain meta-matrices.

Due to the large number of statistics created in reproducing all results across the methodological contingencies, only a part will be directly presented in the supplementary notes and the main text. These are results for with both anaesthesia datasets ordered respectively with the disorders of consciousness dataset. In the main text, results for the 126 whole brain parcellation with the Cambridge collected anaesthesia dataset is presented. More specifically the results presented in the main text and the supplementary notes are with using the aCompCorr (Behzadi, Restom, Liau, & Liu, 2007), with sliding window, the use of Pearson's correlation as a distance metric between connectivity matrices, and with a band pass filter between 0.008 and 0.09. The results where the distance metric, bandpass, denoising, and modality of dynamic functional connectivity matrices creation are varied will be available via internet github links (https://github.com/Peter6789/Meta-Matrix_reproduction), which will be specified for each relevant supplementary note. Below is a display of the way each different combination of pre-processing methods affects the MM for the same individual. These are discussed further below.

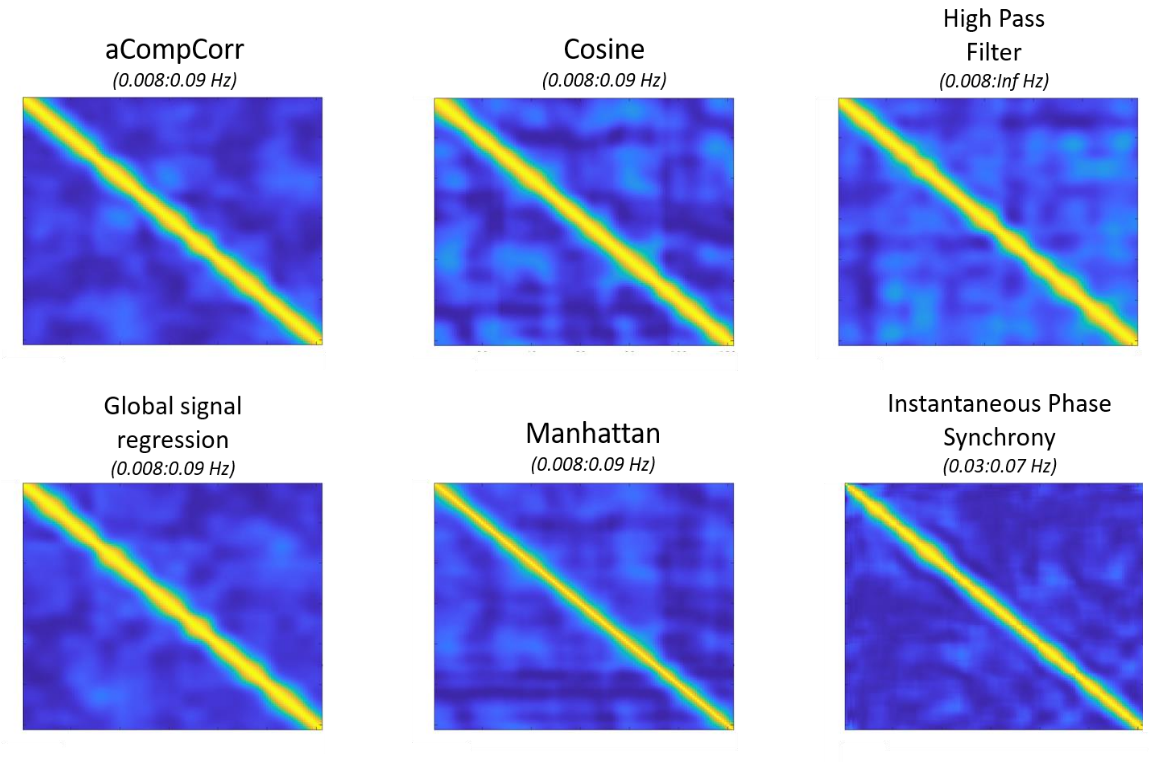

Figure S3. Reproducibility across different pre-processing methods. Shown are MMs for the same individual which were obtained via different methodological contingencies. This included pre-processing of fMRI timeseries data (band pass filtering [Shown in brackets and italics], ACompCorr vs Global Signal regression). We also obtained dynamic functional connectivity matrices via Instantaneous phrase synchrony, as well as Sliding Window (all other MMs beyond Instantaneous Phase synchrony). Finally, we also looked at different methods of examining the relationship between all dynamically varying connectivity patterns (Pearson's, which we used when not stated explicitly, Cosine distance and Manhattan distance [A.K.A. city block]). Where global signal regression is not specified aCompCorr was used .

Noticeable is the difference between differently constructed MMs. It is surprising to find such large differences in the intrinsic dynamics. However, this also increases confidence in the approach, as despite these large differences, effects tended to reproduce across all of them. This indicates that the consciousness relevant dynamic network properties (e.g., variation of speed) remain robust to different ways of pre-processing the data (although when results do not reproduce we state it explicitly in the relevant supplementary notes).

For example, the standard deviation of proximal temporal transitions reproduces univocally across analyses (S4). Similarly subcortical results were significant across all possible contingencies when considered on its own (S8). When inserted in the same ordinal logistic regression with other subsystems (i.e., cerebellum and subcortex), the cortex's effort-to-compress of the distal meta-matric seems to have consistent independent predictive power. We give a summary of how these results reproduce in every relevant supplementary note; but to avoid displaying an impeding number of tables, we direct the reader to a git hub account (https://github.com/Peter6789/Meta-Matrix_reproduction) in which all results are available, should the reader wish to consult them.

Supplementary Note 3

Whole brain results for different parcellations and alternative dataset of the similarity to the proximal temporal similarity model

Ordinal Logistic Regression statistics with similarity to the proximal temporal similarity model as the predictor variable.

We created three models of the Temporal decay of similarity meta-matrix model (TDSM; described in figure 1). One with linearly decreasing similarities between more distant timepoints, and two with exponentially decreasing similarities between distal timepoints (described in methods). We compared each individual's Meta-Matrix (MM), to the TDSMs and the resulting values were inserted as an independent variable in an ordinal logistic regression. To assess whether the proportional odds assumption of the ordinal logistic regression had been violated we performed a Brant's test on all analyses. The predicted variable was the ordered conditions according to presumed level of awareness (i.e. Control Awake> Sedation> MCS>UWS). Presented are Odds Ratios (We standardized [scale in r] all predictor variables to enable comparison across analyses), Confidence intervals range from 2.5% to 97.5%., p-values of the ordinal logistic regression and the p-value of the Brant's test ($p<0.05$ = reject proportional odds assumption). The second table shows the results using the independent anaesthesia dataset collected in London, Ontario, Canada (see methods section).

Table S2. Similarity of PTSM, Ordinal logistic regression results

Odds Ratio Confidence intervals (2.5%:97.5%) P-Value and Brants Test for the linear and exponential TDSMs.

Parcellation	PTSM model	Odds Ratio	Lower C.I.	Higher C.I.	P value	Brants test
**WB126**	**Linear**	2.648205	1.544840	4.949405	0.000457	0.149840
	**Exponential**	3.171873	1.786891	6.222920	0.000127	0.207687
	**Exponential 2**	3.158005	1.806680	5.998699	0.000074	0.095323
**WB553**	**Linear**	5.468442	5.468442	5.468442	0.000019	0.897740
	**Exponental**	3.720424	1.921495	8.411777	0.000228	0.466857
	**Exponential 2**	3.666479	1.957728	7.734874	0.000095	0.327325

Table S3. Similarity of PTSM Ordinal logistic regression results - alternative anaesthesia dataset

Odds Ratio Confidence intervals (2.5%:97.5%) P-Value and Brants Test for the linear and exponential TDSMs

Parcellation	PTSM model	Odds Ratio	Lower C.I.	Higher C.I.	P value	Brants test
**WB126**	**Linear**	1.56968	0.98018	2.51372	0.03028	0.58770
	**Exponential**	2.25488	1.37810	3.68949	0.00061	0.73321
	**Exponential 2**	2.839472	1.698543	4.746774	0.000034	0.851157
**WB553**	**Linear**	2.22212	1.34556	3.66970	0.00091	0.33062
	**Exponental**	3.26926	1.78558	5.98576	0.00006	0.75933
	**Exponential 2**	1.918885	1.238789	2.972355	0.001756	0.427118

Reproducibility across alternative methods for TDSM analyses.

This analysis reproduced reasonably well across methodological contingencies with the exception of when high pass filter was used. Also, assumptions were violated for most analyses when the analysis was done with the instantaneous phase synchrony MMs. Nonetheless, results reproduced when assumptions where not violated. All results are available at https://github.com/Peter6789/Meta-Matrix_reproduction/tree/main/TDSM

Supplementary Note 4

Whole brain results for the proximal temporal complexity for the two anaesthesia datasets ordered with the disorders of consciousness dataset

Shown are results (for ordinal logistic regressions; see methods) for two measures of central tendency (Mean and Median), two measures of distribution breadth (standard deviation and Shannon entropy) and two measures of temporal complexity (effort to compress and sample entropy). These measures were repeated for two whole brain parcellations (described in S1).

Table S4. Proximal temporal measures, Ordinal logistic regression results

Odds Ratio Confidence intervals (2.5%:97.5%), regression coefficient (signed), P-Value and Brants Test for central tendency (mean and median), breadth of distribution (Shannon entropy, standard deviation [STD]) and temporal complexity (effort to compress [ETC] and sample entropy [SampEn]), for proximal transitions.

Measures	Parcellation	Odds Ratio	Lower C.I.	Higher C.I.	Coefficient	P value	Brants test
Mean	126	3.66	9.97	1.75	-1.30	0.00384	1.00
Median	126	4.07	9.78	2.02	-1.40	0.00049	0.85
Mean	553	4.82	16.22	1.98	-1.57	0.00405	0.39
Median	553	4.84	13.04	2.23	-1.58	0.00046	0.82
STD	126	3.47	6.43	2.01	-1.24	0.00002	0.04
Shannon	126	2.84	4.95	1.71	-1.05	0.00010	0.07
STD	553	3.50	6.55	2.02	-1.25	0.00003	0.11
Shannon	553	3.07	5.42	1.84	-1.12	0.00004	0.15
SampEn	126	4.42	2.37	9.08	1.49	0.00001	0.96
ETC	126	2.83	1.66	5.19	1.04	0.00031	0.97
SampEn	553	3.51	1.95	6.93	1.26	0.00009	0.74
ETC	553	2.27	1.37	3.96	0.82	0.00228	0.83

Table S5. proximal temporal measures, Ordinal logistic regression results – Alterative anaesthesia dataset.

Odds Ratio Confidence intervals (2.5%:97.5%), regression coefficient (signed), P-Value and Brants Test for central tendency (mean and median), breadth of distribution (Shannon entropy, standard deviation [STD]) and temporal complexity (effort to compress [ETC] and sample entropy [SampEn]), for proximal transitions.

Measures	Parcellation	Odds Ratio	Lower C.I.	Higher C.I.	Coefficient	P value	Brants test
Mean	126	3.99	9.74	1.96	-1.38	0.00087	1.000
Median	126	3.56	7.56	1.93	-1.27	0.00029	1.000
Mean	553	4.09	10.37	1.96	-1.41	0.00107	1.000
Median	553	3.81	8.45	2.00	-1.34	0.00028	0.007
STD	126	2.03	3.23	1.31	-0.71	0.00203	0.810
Shannon	126	1.70	2.63	1.12	-0.53	0.01399	0.895
STD	553	2.35	3.78	1.50	-0.85	0.00027	0.816
Shannon	553	1.92	2.98	1.26	-0.65	0.00291	0.721
SampEn	126	2.60	1.61	4.39	0.96	0.00016	0.039
ETC	126	2.95	1.83	4.98	1.08	0.00002	0.576
SampEn	553	2.90	1.74	5.11	1.06	0.00010	0.581
ETC	553	2.29	1.44	3.77	0.83	0.00066	0.958

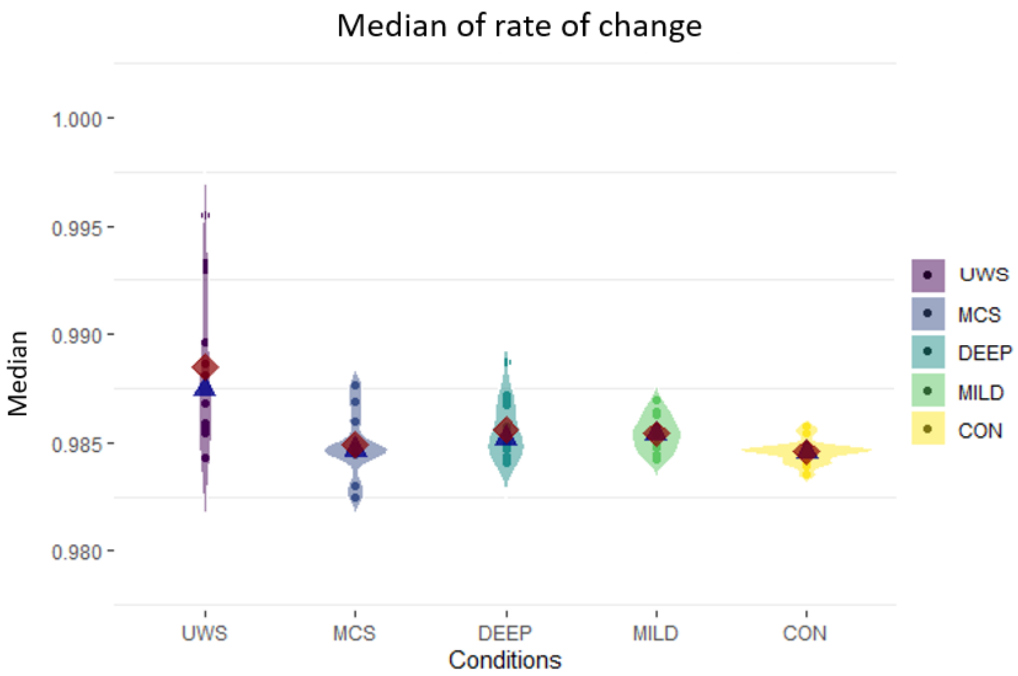
Median of rate of change
Median
1.000
0.995
0.990
0.985
0.980
UWS
MCS
DEEP
MILD
CON
Conditions
UWS
MCS
DEEP
MILD
CON

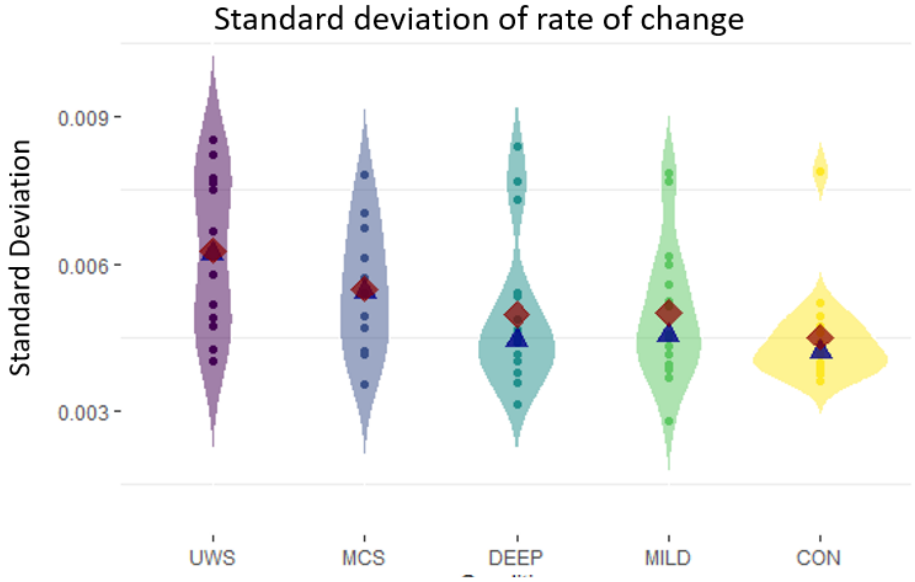
Standard deviation of rate of change
Standard Deviation
0.009
0.006
0.003
UWS
MCS
DEEP
MILD
CON

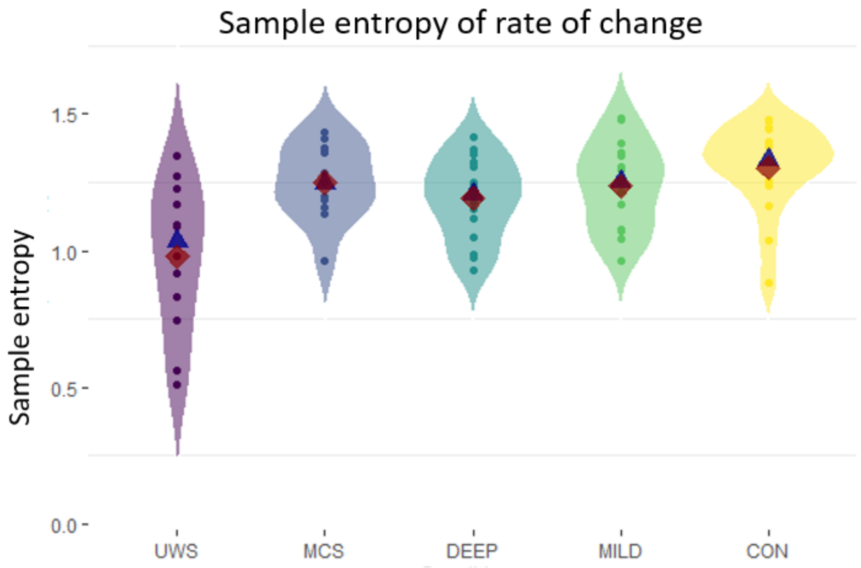
Sample entropy of rate of change
Sample entropy
1.5
1.0
0.5
0.0
UWS
MCS
DEEP
MILD
CON

Figure S4. Results for the proximal temporal analysis for the alternative anaesthesia dataset.

Reproducibility across methodological contingencies for proximal transition analyses.

Results reproduced well across analyses (see S2; all results available from https://github.com/Peter6789/Meta-Matrix_reproduction/tree/main/Proximal_analysis). In particular the standard deviation reproduced unequivocally across methodological contingencies. However, when global signal regression was used there was no replication for the central tendency measures. This may be because the global signal in fact carries important information, particularly in consciousness-relevant conditions, and may be needed to resolve proximal transitions between different network states (Liu, Nalci, & Falahpour, 2017; Tanabe et al., 2020), or may be sensitive to different information that does not permit to distinguish the conditions. Also, the instantaneous phase synchrony did not show replication for the central tendency measures, and only partially reproduced the temporal complexity (ETC and Sample Entropy) measures. Nonetheless results reproduced across all other analyses (see S2), giving a good degree of confidence in the results.

Reproducibility varying the proximal transition time (i.e., 2s, 12s and 26s)

Results reproduced when we looked at different temporal distances (the number of timepoint between two successive connectivity states; described in methods). This was repeated with the two anaesthesia datasets, respectively ordered with the DOC patients, using aCompCorr and sliding window. Replication occurred across all analyses. These are fully accessible in https://github.com/Peter6789/Meta-Matrix_reproduction/tree/main/Proximal_analysis. The relevant CSV files end with *_OTHER_TIMEPOINTS.

Reproducibility of the relationship between the first temporal derivative of proximal network transitions and the standard deviation of such proximal network transitions.

We wanted to confirm the intuition that the variation in the rate of change (indicating increases or decreases in the speed of such a change) corresponded to the amount of change of the rate of change (i.e., the first derivative, which may be interpreted as “acceleration”). Note, the absolute value of the first derivative was used (thus positive and negative acceleration included additively in the same measure). Across participants, we found high correlations between these metrics. For the higher granularity parcellation for the Cambridge anaesthesia dataset (with the DOC dataset), the Rho value was .91, $p<0.0000001$; for the London Ontario dataset the rho value was 0.90, $p<0.0000001$, for the lower granularity parcellation (see S1) and rho=.91, $p<0.00000001$ for the higher granularity parcellation.

Supplementary Note 5

Distal Meta-Matrix (dMM) Characterisations.

Presented is a short discussion of the presumed intrinsic dynamics observable from the dMM in figure 3C. The object of this supplementary note is to give an intuition of the high dimensional state space underlying the dMM. This space is highly reduced by the MM (despite being high dimensional itself, theoretically 1 column = 1 dimension), as a many potential differences in brain connectivity patterns are reduced to one distance metric (in this case Pearson's correlation; see also S2).

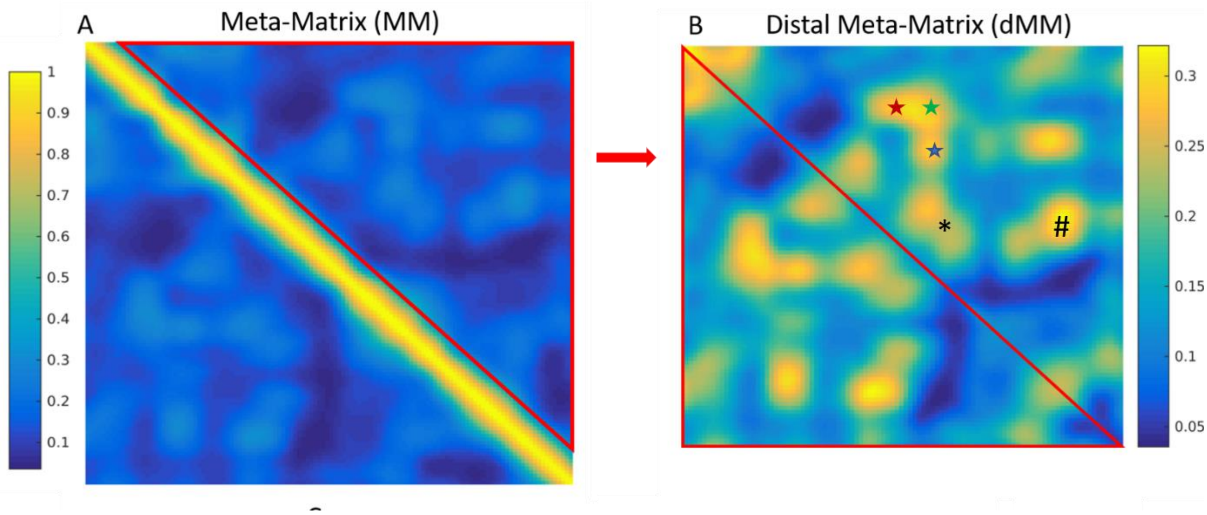

Figure S5. Further characterisations of the dynamics displayed by the dMM.

Patterns may return to relatively similar states via many different trajectories, or two states can be equally similar to a third state, but in different ways. For example, in figure 5B, the red and green stars star show how a connectivity pattern (represented as a row) may be similar to two succeeding states, whilst the following state (just a few rows down) is similar to only one of those states (blue star). In the same figure (Fig. 5) the asterisk shows how states can linearly retrace themselves (high similarities along parallel to the diagonal), but also somewhat retrace themselves "backward in time" (represented by a hash; high similarities along the antidiagonal, orthogonal to the main diagonal). This shows that, despite the great reduction of dimensionality (7875 different connection in low granularity parcellation), complex dynamics relating to a high dimensional state space are observable in the dMM (e.g., retracing forward or in reverse previous patterns; however, the similarities are never too high, as discussed in main text).

Supplementary Note 6

Reproducibility of distal meta-matrix (dMM) results.

On the dMM we tested measures of central tendency, average, and standard deviation (as discussed in the main text). Similarly, to above we inserted each of these measures as independent variables in ordinal logistic regressions. These measures were calculated column wise, but we also repeated the analyses when the whole dMM was vectorised (see below). We also repeated the analysis by varying the number of proximal (autocorrelational) timepoints removed, namely 26s (removed proximal timepoints =13) and 48s (removed proximal timepoints =24).

Table S6. distal meta-matrix measures, Ordinal logistic regression results

Odds Ratio, regression coefficient (signed), P-Value and Brants Test for central tendency (mean and median), breadth of distribution (Shannon entropy, standard deviation [STD]) and temporal complexity (effort to compress [ETC] and sample entropy [SampEn]), for dMM.

Removed proximal timepoints	Measures	Parcellation	Odds Ratio	Coefficient	P value	Brants test
13	Mean	126	1.24	0.22	0.397943	0.000063
13	Median	126	1.53	0.43	0.095554	0.008772
13	Mean	553	1.01	-0.01	0.977343	1.000000
13	Median	553	1.27	0.24	0.370360	1.000000
24	Mean	126	1.55	0.44	0.085705	0.012395
24	Median	126	1.65	0.50	0.050998	0.024407
24	Mean	553	1.32	0.28	0.302119	0.000000
24	Median	553	1.38	0.32	0.231658	0.000000
13	STD	126	1.62	-0.48	0.072510	1.000000
13	Shannon	126	1.16	0.15	0.543851	0.000220
13	STD	553	2.05	-0.72	0.027398	1.000000
13	Shannon	553	1.00	0.00	0.994415	0.000000
24	STD	126	1.12	0.12	0.639856	0.000000
24	Shannon	126	1.56	0.45	0.060354	0.000720
24	STD	553	1.13	-0.12	0.635001	1.000000
24	Shannon	553	1.32	0.27	0.250062	0.001346
13	SampEn	126	4.97	1.60	0.000034	0.558859

13	ETC	126	5.47	1.70	0.000037	0.897740
13	SampEn	553	4.39	1.48	0.000029	0.680429
13	ETC	553	7.71	2.04	0.000001	0.505860
24	SampEn	126	2.38	0.87	0.002477	0.222185
24	ETC	126	2.44	0.89	0.001627	0.382327
24	SampEn	553	1.88	0.63	0.015216	0.118822
24	ETC	553	2.23	0.80	0.003292	0.257586

Table S7. distal meta-matrix measures, Ordinal logistic regression results – Alternative anaesthesia dataset

Odds Ratio, regression coefficient (signed), P-Value and Brants Test for central tendency (mean and median), breadth of distribution (Shannon entropy, standard deviation [STD]) and temporal complexity (effort to compress [ETC] and sample entropy [SampEn]), for dMM.

Removed proximal timepoints	Measures	Parcellation	Odds Ratio	Coefficient	P value	Brants test
13	Mean	126	1.55	0.439316	0.0489187	0.0157670
13	Median	126	1.66	0.505896	0.0232076	0.0334388
13	Mean	553	1.69	0.52236	0.0330233	0.0000000
13	Median	553	1.86	0.621131	0.0100037	0.0000640
24	Mean	126	1.70	0.527954	0.0173644	0.0347312
24	Median	126	1.74	0.552716	0.0131198	0.0446146
24	Mean	553	1.92	0.649898	0.0069506	0.0001021
24	Median	553	1.97	0.676396	0.0048701	0.0004029
13	STD	126	1.01	0.014092	0.9543062	1.0000000
13	Shannon	126	1.55	0.438574	0.0436434	0.0043150
13	STD	553	1.25	-0.21987	0.3587759	1.0000000
13	Shannon	553	1.48	0.394755	0.0746026	0.0000604
24	STD	126	1.42	0.353799	0.1208777	0.0000000
24	Shannon	126	1.74	0.555692	0.0108903	0.0221114
24	STD	553	1.31	0.273138	0.2804540	1.0000000

24	Shannon	553	1.66	0.508706	0.0212957	0.0050339
13	SampEn	126	3.03	1.109268	0.0001559	0.1632076
13	ETC	126	6.54	1.878662	0.0000072	0.5317297
13	SampEn	553	4.03	1.394861	0.0000108	0.9385118
13	ETC	553	6.98	1.942469	0.0000001	0.1821039
24	SampEn	126	2.07	0.728272	0.0027861	0.2180286
24	ETC	126	2.29	0.82728	0.0016780	0.3206690
24	SampEn	553	2.25	0.812926	0.0012297	0.4129960
24	ETC	553	2.18	0.77756	0.0033323	0.5430190

Reproducibility across alternative methods for dMM results.

Given the absence of significant effects in the central tendency and distribution measures we did not investigate these measures in the alternative analyses (varying methodological contingencies; see S2). Although the alternative anaesthesia dataset seemed to given some significant results in these measures (table 7), the brant's tests were significant (proportional odds violated), and thus the analyses were discounted.

Results did not reproduce with global signal regression (see S4 for potential explanations). Furthermore, with instantaneous phase synchrony only the lower granularity parcellation tended to be significant. However, it should be noted, as shown below (S9), the cortex for these measures (ETC and SampEn of dMM) remains a significant independent predictor with these methodological contingencies. The rest of the results reproduced univocally. Full results can be found at https://github.com/Peter6789/Meta-Matrix_reproduction/tree/main/dMM_analysis.

Supplementary Note 7

Results for proximal and distal measures when inserted into the same ordinal logistic regression.

Here we explore to what extent the two aspects (termed, proximal and distal) of the MM we looked at, can retain independent variance. To ensure multicollinearity was not violated, but also to increase the change of not violating the proportional odds assumption, we inserted only the measures with the higher odds ratios in the same ordinal logistic regression (as covariates). These two measures where the sample entropy of the proximal transitions and the effort-to-compress of the dMM.

Table S8. Sample entropy of proximal timepoint transition and effort-to-compress of dMM, inserted in the same ordinal logistic regression

Odds Ratio (OR), regression coefficient (signed), P-Value and Brants Test for ETC of dMM (represented by "dMM"), and the sample entropy of proximal values (represented by "Proximal") when inserted into the same ordinal logistic regression.

Networks	OR_dMM	Coefficient dMM	P value dMM	OR Proximal	Coefficient Proximal	P-Value Proximal	Brants test
126	4.23	1.44	0.00158	3.67	1.30	0.0009	0.98
553	6.04	1.80	0.00002	2.17	0.77	0.0319	0.78

Table S9. Sample entropy of proximal timepoint transition and effort-to-compress of dMM, inserted in the same ordinal logistic regression – Alternative anaesthesia dataset.

Odds Ratio (OR), regression coefficient (signed), P-Value and Brants Test for ETC of dMM (represented by "dMM"), and the sample entropy of proximal values (represented by "Proximal") when inserted into the same ordinal logistic regression.

Networks	OR_dMM	Coefficient dMM	P value dMM	OR Proximal	Coefficient Proximal	P-Value Proximal	Brants test
126	5.65	1.73	0.00025	1.25	0.22	0.50	0.43
553	5.84	1.76	0.00002	1.32	0.28	0.43	0.10

Reproducibility across alternative methods of unique predictive variance of proximal and distal measures when inserted as covariates in the same ordinal logistic regression.

Whilst with global signal regression and high pass filter, only the proximal measures were significant; in the alternative anaesthesia dataset (with aCompCorr and bandpass filter between 0.008 and 0.09; shown in table 9) with the use of instantaneous phase synchrony (IPS), the majority of dMM analyses were significant (except lower granularity for IPS, p=0.06). When the Manhattan distance measure was used, both measures were significant, only in the higher granularity parcellation, whilst only the proximal measure was significant in the lower granularity parcellation. With the cosine distance both

measures were significant. Full results can be found at https://github.com/Peter6789/Meta-Matrix_reproduction/tree/main/Proximal_and_Distal_in_same_OLR. This indicates that the degree of independent predictive power of each measure varies as a function of methodological contingencies. Thus, given the relative symmetry between proximal and distal results, we are not able to conclude whether either one has more predictive power, or whether they have independent explanatory power.

Reproducibility of correlations between the different measures across parcellation granularities and the alternative anaesthesia dataset

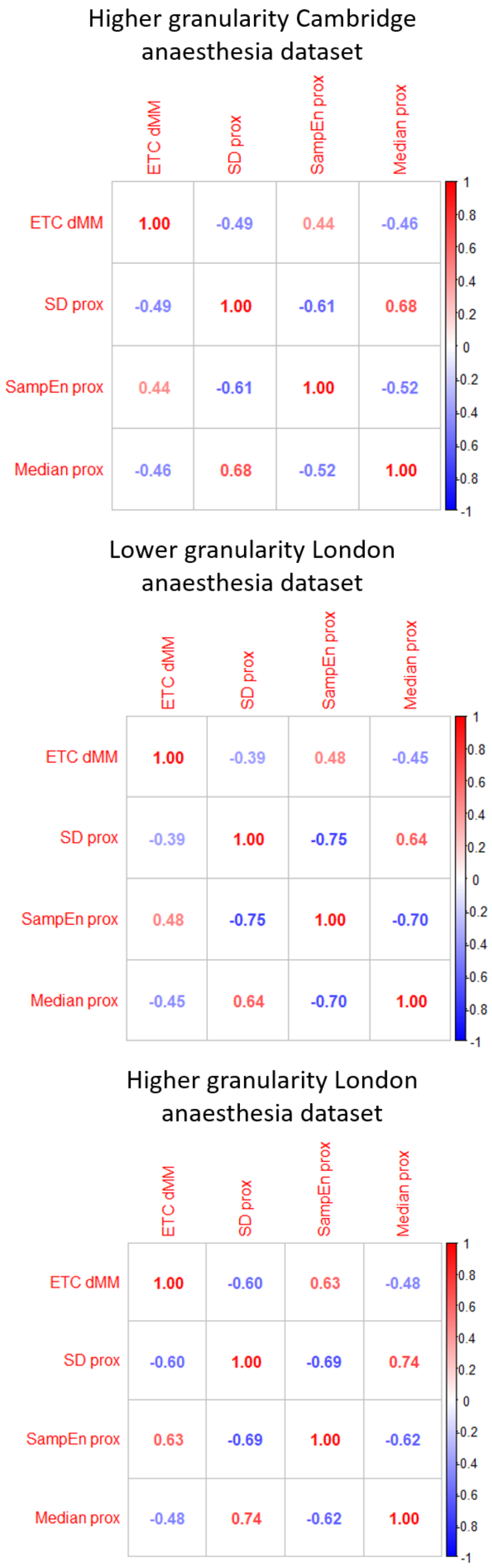

Figure S6. Spearman correlation between all variables for the higher and lower granularities across alternative anaesthesia datasets.

Supplementary Note 8

Results for proximal and distal measures for individual subsystems (cortex, subcortex and cerebellum) when inserted into ordinal logistic regressions separately.

Table S10. Results for proximal and distal measures for the cortex, subcortex and cerebellum. – **Alternative anaesthesia dataset.**

Presented are Odds Ratios, and P-Values for each measure (rows) and each system (columns). ETC= effort-to-compress; STD= standard deviation.

Measures	Cortex		Subcortex	
	Odds Ratio	P-Value	Odds Ratio	P-Value
Proximal SampEn	2.47	0.0005764	2.85	0.0000004
Proximal STD	2.13	0.0009909	2.31	0.0000001
Proximal Median	2.99	0.0008657	4.68	0.0000006
dMM ETC	8.69	0.0000001	3.61	0.0000005

Note: Insufficient coverage of cerebellum in the alternative anaesthesia dataset.

Table for the Cambridge anaesthesia dataset presented in main text.

Reproducibility across methodological contingencies.

The cortex did not reproduce well with global signal regression (specifically, sample entropy and median of proximal transitions), whilst cerebellar results were similar to those presented in the main text. See S4 for possible explanations. All other analyses (see S2) reproduced results. Subcortical results were unequivocally reproduced across analyses. Full results are available at https://github.com/Peter6789/Meta-Matrix_reproduction/tree/main/Subsystems_in_separate_OLRs.

Supplementary Note 9

Results for proximal and distal measures for individual subsystems (cortex, subcortex and cerebellum) when inserted into the same ordinal logistic regression as covariates.

Table S11. Results for proximal and distal measures for the cortex, subcortex and cerebellum when inserted into the same ordinal logistic regression.

Presented are Odds Ratios, and P-Values for each measure (rows) and each system (columns) when inserted into the same ordinal logistic regression. ETC= effort-to-compress; STD= standard deviation.

	Cortex			Subcortex			Cerebellum			
Measures	OR	Coef	P-Value	OR	Coef	P-Value	OR	Coef	P-Value	Brant’s test
Proximal SampEn	1.77	0.57	0.1214	1.25	0.22	0.51	1.67	0.51	0.14	0.58
Proximal STD	1.85	-0.61	0.3649	1.25	0.22	0.67	2.51	-0.92	0.11	0.13
Proximal Median	1.16	-0.15	0.8329	1.57	-0.45	0.48	1.84	-0.61	0.19	0.12
dMM ETC	3.72	1.31	0.0003	1.54	0.43	0.20	1.19	0.18	0.60	0.59

Table S12. Results for proximal and distal measures for the cortex, subcortex and cerebellum when inserted into the same ordinal logistic regression- Alternative anaesthesia dataset.

Presented are Odds Ratios, and P-Values for each measure (rows) and each system (columns) when inserted into the same ordinal logistic regression. ETC= effort-to-compress; STD= standard deviation.

	Cortex			Subcortex			
Measures	OR	Coef	P-Value	OR	Coef	P-Value	Brant’s test
Proximal SampEn	1.37	0.32	0.398725	2.28	0.82	0.03	0.33
Proximal STD	1.12	-0.12	0.829144	2.07	-0.73	0.20	0.05
Proximal Median	1.39	0.33	0.561510	6.00	-1.79	0.002	1.00
dMM ETC	8.76	2.17	0.000006	1.01	-0.01	0.98	0.13

Note: Insufficient coverage of cerebellum in the alternative anaesthesia dataset.

Reproducibility across methodological contingencies.

The only consistent result was the effort-to-compress in the cortex (although with instantaneous phase synchrony p=0.06). Whilst cerebellar and subcortical subsystems seem to retain some independent variance in some analyses, this did not reproduce across methodological contingencies (see S2 for descriptions). Full results are available at https://github.com/Peter6789/Meta-Matrix_reproduction/tree/main/Subsystems_in_same_OLR.

Supplementary Note 10

Complexity of relationship between structural and dynamic functional connectivity.

For this analysis, the dynamic functional connectivity matrices for each individual were thresholded to match the number of connections found with the diffusion tensor imaging analysis to thus ensure comparability. There were then vectorised and correlated thus obtaining a vector of similarities between the structural and functional connectivity that varied across time. On this vector the complexity measures were calculated (Sample Entropy and Effort-to-compress) which were subsequently used as predictor variables in the ordinal logistic regressions. Since the DOC and the Control Datasets had different number of timepoints, we chose a subset of the longer dataset (295 timepoints) to match the shorter one (131 timepoints), to ensure results were not dependent on the different amount of data available between conditions. In this case it is presented in the table as SampEn-r (reduced) and ETC-r (effort-to-compress – reduced). The ETC analysis was not performed using all the DOC timepoints because it is very sensitive to the length of the vector to be compressed, and therefore not comparable between conditions unless these have similar numbers of timepoints (i.e., ETC-r).

Due to function failure of the Brant's test the proportional odds assumption was tested by creating a generalised linear model with and without proportional odds assumption and testing is they are significantly different. NAN values indicate that the fit of the non-parallel model resulted either in a 1 or a 0, and therefore is not usable. In this case, the alternative analyses should be used for interpretation. The p-value for this is presented in the last column under assumption test.

The DOC DTI dataset was acquired with two different acquisitions methods (described in the methods section). To ensure that the two different acquisitions did not affect results, we ran a confirmatory analysis excluding participants that had been acquired with the 12-direction method. This left with eight participants for each condition (MCS; UWS) that hadn't been acquired with the 63 direction method. Below are the results for this confirmatory analysis

Table S13. Complexity of the relationship between dynamic functional and structural connectivity - Ordinal logistic regressions.

Odds Ratios, Confidence intervals (2.5%:97.5%), P-Value and Parallel & non-parallel model comparison for the whole brain parcellations (126 & 553 regions), the cortical parcellations (100 & 400 regions), the subcortex and the cerebellum.

Parcellation	Entropy measure	Odds Ratio	Lower C.I.	Higher C.I.	P value	Assumption test
**WB126**	**SampEn**	2.957317	1.393049	6.278113	0.002379	0.121154
	**SampEn-r**	2.151162	1.130210	4.094369	0.009832	0.119661
	**ETC-r**	1.799330	0.967223	3.347303	0.031820	0.940105
**WB553**	**SampEn**	5.157884	2.067199	12.869473	0.000219	0.810988
	**SampEn-r**	3.247688	1.512874	6.971815	0.001255	NaN
	**ETC-r**	1.71716	0.923313	3.193556	0.043827	0.756838
**Cor1**	**SampEn**	5.425423	2.050584	14.354550	0.000329	0.160355
	**SampEn-r**	2.976889	1.433132	6.183566	0.001723	0.868415
	**ETC-r**	2.029420	1.070494	3.847332	0.015053	0.510462
**Cor4**	**SampEn**	3.936728	1.757401	8.818604	0.000434	0.580164
	**SampEn-r**	2.954336	1.471344	5.932058	0.001161	0.167070
	**ETC-r**	2.266940	1.215758	4.227005	0.005019	0.124635
**Subcortex**	**SampEn**	3.837187	1.729448	8.513700	0.000471	0.048873
	**SampEn-r**	7.965344	2.662586	23.828980	0.000103	0.630322
	**ETC-r**	3.018306	1.437153	6.339039	0.001762	0.621395
**Cerebellum**	**SampEn**	3.360443	1.499453	7.531128	0.001621	0.323433
	**SampEn-r**	2.355980	1.196941	4.637357	0.006564	0.662889
	**ETC-r**	1.710466	0.942567	3.103962	0.038748	0.782862

Table S14. Complexity of the relationship between dynamic functional and structural connectivity - Ordinal logistic regressions. NB: 63 Direction acquisition data only.

Odds Ratios, Confidence intervals (2.5%:97.5%), P-Value and Parallel & non-parallel model comparison for the whole brain parcellations (126 & 553 regions), the cortical parcellations (100 & 400 regions), the subcortex and the cerebellum.

Parcellation	Entropy measure	Odds Ratio	Lower C.I.	Higher C.I.	P value	Assumption test
**WB126**	**SampEn**	2.738186	1.226889	6.111116	0.006962	0.495133
	**SampEn-r**	2.451915	1.189389	5.054602	0.007551	0.002296
	**ETC-r**	1.64846	0.861827	3.153093	0.065445	NaN
**WB553**	**SampEn**	5.603857	1.872774	16.768287	0.001028	0.482302
	**SampEn-r**	4.619301	1.813022	11.769270	0.000671	0.802431
	**ETC-r**	2.02779	0.988292	4.160637	0.02694	0.229578
**Cor1**	**SampEn**	6.419385	1.847255	22.307963	0.001719	0.120797
	**SampEn-r**	3.338921	1.413554	7.886784	0.002987	0.676726
	**ETC-r**	1.79249	0.899532	3.571888	0.048558	0.411966
**Cor4**	**SampEn**	4.928692	1.664981	14.589965	0.001984	0.804876
	**SampEn-r**	3.996111	1.647570	9.692399	0.001090	0.238633
	**ETC-r**	2.26371	1.108733	4.621836	0.012436	0.408835
**Subcortex**	**SampEn**	4.875621	1.777856	13.370986	0.001042	0.909377
	**SampEn-r**	7.534388	2.171974	26.136130	0.000731	0.899040
	**ETC-r**	3.78274	1.472335	9.718672	0.002859	0.176704
**Cerebellum**	**SampEn**	2.155642	1.042055	4.459258	0.019179	0.335637
	**SampEn-r**	2.019571	0.997312	4.089656	0.025439	0.207257
	**ETC-r**	1.66794	0.872698	3.187851	0.060811	0.632654

Supplementary Note 11

Complexity of relationship between structural and dynamic functional connectivity- reproducibility across different distance metrics.

We sought to reproduce the structure to function dynamic complexity results using alternative distance metrics (namely Cosine and Manhattan/City-Block distance). Specifically, we applied these metrics to quantify the similarity/distance between the structural and functional connectivity matrices. These metrics were therefore used as an alternative to Pearson's correlation, analogously to what is shown in S2. We reproduced these results using only sample entropy (without reducing timepoints, given the replications above, e.g., S10) and for the subsystems specifically (cortex, subcortex and cerebellum). Interestingly, as shown below (tables 15, and 16), the Cosine distance reproduced all results, whilst the Manhattan distance reproduced only subcortical results.

Table S15. Complexity of the relationship between dynamic functional and structural connectivity when Manhattan distance is used- Ordinal logistic regressions.

Odds Ratios, Confidence intervals (2.5%:97.5%), P-Value for the cortical parcellations (100 & 400 regions), the subcortex and the cerebellum.

Parcellation	Entropy measure	Odds Ratio	Lower C.I.	Higher C.I.	P value
**Cor1**	**SampEn**	1.413804	0.384942	1.266468	0.124394
**Cor4**	**SampEn**	1.550781	0.346502	1.159202	0.073906
**Subcortex**	**SampEn**	2.233708	0.220534	0.833672	0.008157
**Cerebellum**	**SampEn**	1.404924	0.385235	1.267238	0.128104

Table S16. Complexity of the relationship between dynamic functional and structural connectivity when Cosine distance is used- Ordinal logistic regressions.

Odds Ratios, Confidence intervals (2.5%:97.5%), P-value for the cortical parcellations (100 & 400 regions), the subcortex and the cerebellum.

Parcellation	Entropy measure	Odds Ratio	Lower C.I.	Higher C.I.	P value
**Cor1**	**SampEn**	5.349279	0.062497	0.440521	0.000328
**Cor4**	**SampEn**	3.91548	0.104002	0.532645	0.00044
**Subcortex**	**SampEn**	3.854012	0.109222	0.53434	0.000378
**Cerebellum**	**SampEn**	2.580203	0.187238	0.741368	0.003157

Reproducibility of the independent predictive power of the cortex, subcortex and cerebellum with the alternative distance metrics.

We inserted the sample entropy of the dynamic's similarity (inverse of distance) between the structural and functional connectivity for the different subsystems in the same ordinal logistic regression as covariates.

Table S17. Reproducibility of independent predictive power of the cortex, subcortex and cerebellum when inserted as covariates in the same ordinal logistic regression. Distance metric is Manhattan.

Parcellation	Entropy measure	Odds Ratio	P value
**Cortex**	**SampEn**	1.10	0.39
**Subcortex**	**SampEn**	2.16	0.02
**Cerebellum**	**SampEn**	1.37	0.18

Table S18. Reproducibility of independent predictive power of the cortex, subcortex and cerebellum when inserted as covariates in the same ordinal logistic regression. Distance metric is Cosine.

Parcellation	Entropy measure	Odds Ratio	P value
**Cortex**	**SampEn**	2.93	0.008
**Subcortex**	**SampEn**	2.91	0.005
**Cerebellum**	**SampEn**	1.93	0.06
