# Supplementary material for: The complexity of the stream of consciousness
Source: Commun Biol. 2022 Nov 3;5:1173. doi: 10.1038/s42003-022-04109-x (PMC9633704; doi:10.1038/s42003-022-04109-x)
Supplement: Supplementary file 3 — nr-reporting-summary [file 42003_2022_4109_MOESM3_ESM.pdf]

nature portfolio

Corresponding author(s): Emmanuel A Stamatakis

Last updated by author(s): 15/08/22

Reporting Summary

Nature Portfolio wishes to improve the reproducibility of the work that we publish. This form provides structure for consistency and transparency in reporting. For further information on Nature Portfolio policies, see our Editorial Policies and the Editorial Policy Checklist.

Statistics

For all statistical analyses, confirm that the following items are present in the figure legend, table legend, main text, or Methods section.

n/a	Confirmed	
☐	☒	The exact sample size (*n*) for each experimental group/condition, given as a discrete number and unit of measurement
☐	☒	A statement on whether measurements were taken from distinct samples or whether the same sample was measured repeatedly
☐	☒	The statistical test(s) used AND whether they are one- or two-sided<br>*Only common tests should be described solely by name; describe more complex techniques in the Methods section.*
☐	☒	A description of all covariates tested
☐	☒	A description of any assumptions or corrections, such as tests of normality and adjustment for multiple comparisons
☐	☒	A full description of the statistical parameters including central tendency (e.g. means) or other basic estimates (e.g. regression coefficient) AND variation (e.g. standard deviation) or associated estimates of uncertainty (e.g. confidence intervals)
☐	☒	For null hypothesis testing, the test statistic (e.g. *F*, *t*, *r*) with confidence intervals, effect sizes, degrees of freedom and *P* value noted<br>*Give P values as exact values whenever suitable.*
☒	☐	For Bayesian analysis, information on the choice of priors and Markov chain Monte Carlo settings
☒	☐	For hierarchical and complex designs, identification of the appropriate level for tests and full reporting of outcomes
☐	☒	Estimates of effect sizes (e.g. Cohen's *d*, Pearson's *r*), indicating how they were calculated

Our web collection on statistics for biologists contains articles on many of the points above.

Software and code

Policy information about availability of computer code

Data collection	MRI software and hardware (Siemens) were used to collect the data
Data analysis	Coding languages used were Matlab, R and BASH Some codes were created ad-hoc for the study. Preprocessing was performed using SPM (https://www.fil.ion.ucl.ac.uk/spm/), time-series extraction using CONN (https://web.conn-toolbox.org/). Sample Entropy and Effort to Compress codes were taken from the relevant papers. MRtrix3 (https://www.mrtrix.org/) and DSI studio (www.dsi-studio.labsolver.org). was used for diffusion weighted imaging processing. Ordinal Logistic Regressions were performed using the MASS R toolbox (polr function, https://www.rdocumentation.org/packages/MASS/versions/7.3-53/topics/polr).

For manuscripts utilizing custom algorithms or software that are central to the research but not yet described in published literature, software must be made available to editors and reviewers. We strongly encourage code deposition in a community repository (e.g. GitHub). See the Nature Portfolio guidelines for submitting code & software for further information.

Data

Policy information about availability of data

All manuscripts must include a data availability statement. This statement should provide the following information, where applicable:

- Accession codes, unique identifiers, or web links for publicly available datasets
- A description of any restrictions on data availability
- For clinical datasets or third party data, please ensure that the statement adheres to our policy

The London Ontario Dataset is available (https://openneuro.org/datasets/ds003171). Due to the clinical nature of the data, this will be made available upon reasonable request to the corresponding author.

Field-specific reporting

Please select the one below that is the best fit for your research. If you are not sure, read the appropriate sections before making your selection.

☒ Life sciences ☐ Behavioural & social sciences ☐ Ecological, evolutionary & environmental sciences

For a reference copy of the document with all sections, see nature.com/documents/nr-reporting-summary-flat.pdf

Life sciences study design

All studies must disclose on these points even when the disclosure is negative.

Sample size

There were four datasets used, of which one for replication. Cambridge anaesthesia dataset n=18; disroders of consciousness dataset n=23; London Ontario dataset; Control DWI dataset n=18

Data exclusions

For the Cambridge anaesthesia dataset 7 participants were excluded. This was due to incomplete data in the cortex and anaesthesic procedure failure. The disorders of consciousness data included were selected out of a large clinical cohort (n=71). These were selected due to relatively intact neuroanatomy via extensive visual assessment. A relatively intact anatomy enables a comparison with healthy subjects. For the London Ontario dataset 3 participants were excluded due to equipment malfunction or impairments with anaesthetic procedure. For the control diffusion weighted imaging dataset, 5 participants were excluded due to incomplete data or equipment malfunction.

Replication

We conducted extensive replications. Firstly, we reproduced all analyses with lower and higher granularity brain parcellations. Secondly we also reproduced all analyses (not including diffusion weighted imaging based results), using an independent anaesthesia dataset (i.e., London Ontario dataset). For the proximal time similarity analysis, we created three models of comparison to assess robustness of results. One with linearly decreasing values (of proximal time point similarities) and two exponentially decreasing (slowly decreasing and rapidly decreasing). Results showed convergence (in cerebellum and subcortex either ordinal logistic regression had assumption violations or were not significant). For the complexity of distal similarity time points we conducted four replication analyses. Firstly we varied the number of proximal time point similarities to be removed by choosing values corresponding to the whole temporal sliding window (used for dynamic connectivity calculation n=24) and one time point more than half of the sliding window (n=13). Then we also used different complexity calculation measures, both based on the notion of Kolmogorov complexity, these were sample entropy and effort to compress. All results replicated except for the subcortex when 24 time points were removed. Nonetheless this showed a trend. The structural to functional dynamic similarity results were reproduced in several ways. Firstly, as above we used both effort to compress and sample entropy. Sample entropy was ran twice, once with the same number of time points between datasets and once with unequal numbers (this measure being robust for unequal time series lengths, which is not the case for effort to compress). Secondly, given that two different diffusion weighted imaging acquisition schemes were used we ran a confirmatory analysis excluding patients (n=7) that did not have images with parameters that matched those of the control's images. Furthermore, following the reviewers comments we reproduced all results pertaining to the meta-matrix using instantaneous phase synchrony, a high pass filter (instead of bandpass), global signal regression (instead of component correction method) and different distance metrics instead on Pearson's correlation in constructing the meta-matrix (namely Cosine and Manhattan distance). We also used different temporal intervals (1, 6 and 12 TRs) to investigate the replication of results indicating consciousness is associated with specific properties of short term network dynamics. Further more we also replicated the distal meta-matrix results by vectorizing the whole matrix rather than calculating complexity measures on each column individually. Finally we also applied different distance metrics (cosine and manhattan) in the structure-to-function dynamic complexity analysis. Please see the rebuttal letter or the supplementary materials for more details.

Randomization

Randomisation was possible for one dataset: the Cambridge anaesthesia dataset, as this was conducted in Addenbrooke's hospital. The order of sedation level was randomised. This was not possible with the London Ontario dataset as acquisition was not performed in a hospital (two anesthetists and one anaesthetic nurse being present) and therefore randomisation was not performed so as to ensure participant safety.

Blinding

Given the clinical nature of the data used, blinding was not possible to ensure participant safety.

Reporting for specific materials, systems and methods

We require information from authors about some types of materials, experimental systems and methods used in many studies. Here, indicate whether each material, system or method listed is relevant to your study. If you are not sure if a list item applies to your research, read the appropriate section before selecting a response.

Materials & experimental systems

n/a	Involved in the study	
☒	☐	Antibodies
☒	☐	Eukaryotic cell lines
☒	☐	Palaeontology and archaeology
☒	☐	Animals and other organisms
☐	☒	Human research participants
☐	☒	Clinical data
☒	☐	Dual use research of concern

Methods

n/a	Involved in the study	
☒	☐	ChIP-seq
☒	☐	Flow cytometry
☐	☒	MRI-based neuroimaging

Human research participants

Policy information about studies involving human research participants

Population characteristics	Cambridge anaesthesia: 9/18 (age 19-52). London ontario : 13/25 males; (age 18-40); Diffusion weighted imaging dataset mean age 34 SD 10.7. For the disorders of consicousness dataset 15/23 patients were male, mean age 39.67.
Recruitment	The DOC data was a clinical sample. These were selected due to relatively intact anatomy. This may have biased the sample in terms of extrapolating to the entire DOC population. However, including more participants may have biased the analyses in other ways. Furthermore our question was regarding the neural correlates of consciousness, rather than this clinical population per say. This is a notoriously difficult population to study. Cambridge anaesthesia participants and DWI control participants were recruited via poster advertisements placed in the hospital.
Ethics oversight	London Ontario Anaesthesia dataset: Health Sciences Research Ethics Board and Psychology Research Ethics Board of Western University. Cambirdge Anaestheisa dataset and control DWI dataset: Cambridgeshire 2 Regional Ethics committee. For the DOC dataset: National Research Ethics Service.

Note that full information on the approval of the study protocol must also be provided in the manuscript.

Clinical data

Policy information about clinical studies

All manuscripts should comply with the ICMJE guidelines for publication of clinical research and a completed CONSORT checklist must be included with all submissions.

Clinical trial registration	*Provide the trial registration number from ClinicalTrials.gov or an equivalent agency.*
Study protocol	*Note where the full trial protocol can be accessed OR if not available, explain why.*
Data collection	*Describe the settings and locales of data collection, noting the time periods of recruitment and data collection.*
Outcomes	*Describe how you pre-defined primary and secondary outcome measures and how you assessed these measures.*

Magnetic resonance imaging

Experimental design

Design type	Resting state.
Design specifications	London Ontario dataset= 256 functional volumes (8 minutes) per anaesthetic state. DOC dataset 300 volumes (10 minutes); Cambridge anaestesia dataset 150 volumes (5 minutes) per anaesthetic state
Behavioral performance measures	N/A

Acquisition

Imaging type(s)	Functional, Structural, Diffusion
Field strength	3
Sequence & imaging parameters	Cambridge anaethesia dataset= 32 descending interleaved oblique axial slices with an interslice gap of 0.75 mm and an in plane resolution of 3 mm. The field of view was 192x192, Repetition time and acquisition time was 2 seconds whilst the echo time was 30 ms and flip angle 78. Echo-planar sequence. T1-weighted structural images with 1mm resolution were obtained using an MPRAGE sequeunce with TR= 2250 ms , TI – 900ms , TE = 2.99 ms flip angle= 9 degrees. London Ontario Dataset = : slices=33, 25% inter-slice gap resolution 3mm isotropic; TR=2000ms; TE=30ms; flip-angle=75 degrees; matrix=64x64. Order-of-acquisition was bottom-up interleaved. Echo-planar sequence. The anatomical high-resolution T1 weighted images (32-channel coil 1mm isotropic voxels) were acquired using a 3D MPRAGE sequence with TA=5mins, TE =4.25ms, matrix=240x256, 9 degrees FA. DOC dataset= TR=2s using a siemens TRIO 3T scanner. The functional images were acquired using an echo planar sequence. Parameters include: 3x3x3.75mmm resolution, TR/TE = 2000ms/30ms, 78 degrees FA. Anatomical images T1-weighted images were acquired using a repetition time of 2300ms, TE=2.47ms, 150 slices with a cubic resolution of 1 mm. For DWI controls: TR=2000 ms, TE=30 ms, flip angle=78º, FOV read=192 mm, voxel size=3.0x3.0x3.0 mm, slices per volume=32; Echo-planar imaging, matrix size 64x64. For T1 images (MPRAGE sequence) structural scan TR=2300ms, TE=2.98 ms, TA=9.14 min, flip angle=9°, field of view read=256 mm, voxel size=1.0x1.0x1.0mm, slices=176 with magnetization-prepared 180 degrees radio-frequency pulses and rapid gradient-echo.
Area of acquisition	Whole brain. If cortex was excluded, data was removed from analyses. London ontario dataset did not have complete cerebellum coverage, as did one DOC patient. These data were excluded from cerebellar analyses.

Diffusion MRI ☒ Used ☐ Not used

Parameters	12 non-colinear directions with 5 b=0 and 5 b-values that ranged from 340 to 1590 s/mm2 & 63 directions with a b-value of 1000 s/mm2

Preprocessing

Preprocessing software	For functional images: SPM12's functions of slice-timing correction (reference slice=no. 17) and movement correction by realignment to mean functional image. No smoothing performed. For structural images, participant-specific gray matter, cerebral spinal fluid and white matter masks were also created using the segmentation function of SPM12. Resting state and time series relevant extraction was performed by CONN using default parameters (and inclusion of ART toolbox).<br>for DWI: MRtrix3 dwipreproc dwibiascorrect dwi2mask) . FSL's eddy & BET command (participant specific values were entered and assessed via visual inspection). DSI studio package's q-space diffeomorphic reconstruction (QSRD) and SPM nonlinear registration function.
Normalization	DWI and structural: SPM nonlinear registration function to TPM image (MNI). For functionals non-linear normalisation of functional images to EPI template directly as this gave best results.
Normalization template	MNI template as in SPM's "TPM.nii" for structural and DTI data. For functional data the EPI normalized template that is included in SPM was used.
Noise and artifact removal	Removing the first 5 scans. Inclusion of principle components (n=5) from white matter and cerebral spinal fluid, and moment parameters as regressors of no interest. Band pass filtering 0.008 to 0.9 Hz (CONN default). We also ran analysis with global signal regression and using a high pass filter (0.008 to inf).
Volume censoring	We extensively visually assessed the data. We also used ART toolbox as implemented in CONN to deweight scans which were were deemed outliers in "denoising" step regression.

Statistical modeling & inference

Model type and settings	We modeled individual participant's data as a multivariate intrinsically defined dynamic space. This modeling and the testing against proximal time similarity models are somewhat analogous to representational similarity analysis, although quite removed from it. Values describing this dynamic space were extracted and inserted into an ordinal logistic regression for inferential analyses.
Effect(s) tested	Predicting ordinal levels of awareness (control awake, sedation and disorders of consciousness) using properties of the intrinsic dynamic space calculated via resting state.

Specify type of analysis: ☐ Whole brain ☐ ROI-based ☒ Both

Anatomical location(s)	Whole brain, but also subdivisions into cortex, subcortex (not including cerebellum and brain stem) and finally the cerebellum
Statistic type for inference (See Eklund et al. 2016)	Ordinal logistic regression. Used as it had assumptions that were suitable to these analyses. Ordinal logistic regression. Used as it had assumptions that were suitable to these analyses. Proportional odds assumption tested via Brant's test.
Correction	FWE correction used.

Models & analysis

n/a	Involved in the study
☐	☒ Functional and/or effective connectivity
☒	☐ Graph analysis
☐	☒ Multivariate modeling or predictive analysis

Functional and/or effective connectivity	Pearson Correlation
Multivariate modeling and predictive analysis	Similarity of intrinsic dynamic space ("meta-matrix") to proximal time similarity models. Average, variation and complexity of short term transitions (i.e., moment to moment distances in network dynamics). Average complexity of meta-matrix columns (corresponding to timepoints), and complexity of structural-functional dynamic similarity. Odds ratio and p-values were the principle statistics used for evaluation.
